# Growth Media Induces Variation in Cell Wall Associated Gene Expression in *Arabidopsis thaliana* Pollen Tube

**DOI:** 10.3390/plants2030429

**Published:** 2013-06-25

**Authors:** Mário Luís da Costa, Luís Gustavo Pereira, Sílvia Coimbra

**Affiliations:** BioFIG, Center for Biodiversity, Functional and Integrative Genomics, Biology Department, Faculty of Sciences, University of Porto, Rua do Campo Alegre s/n 4169-007 Porto, Portugal; E-Mails: mariocost@fc.up.pt (M.L.D.C.); lcpereir@fc.up.pt (L.G.P.)

**Keywords:** *Arabidopsis thaliana*, pollen tube, pollen tube growth media, Arabinogalactan proteins, real-time PCR

## Abstract

The influence of three different pollen germination media on the transcript profile of Arabidopsis pollen tubes has been assessed by real-time PCR on a selection of cell wall related genes, and by a statistical analysis of microarray Arabidopsis pollen tube data sets. The qPCR assays have shown remarkable differences on the transcript levels of specific genes depending upon the formulation of the germination medium used. With the aid of principal component analysis performed on existing microarray data, a subset of genes has been identified that is more prone to produce diverging transcript levels. A functional classification of those genes showed that the clusters with higher number of members were those for hydrolase activity (based in molecular function) and for cell wall (based in cellular component). Taken together, these results may indicate that the nutrient composition of the pollen germination media influences pollen tube metabolism and that caution must be taken when interpreting transcriptomic data of pollen tubes.

## 1. Introduction

The importance of double fertilization for the flowering plant life cycle makes the study of all the steps involved in the process of crucial importance. Pollen grains land on compatible stigmas and germinate growing a tube characterized by an apical type of growth, penetrating the extracellular matrix of different parts of the pistil and finally reaching the embryo sac. Pollen tubes have always been considered one of the best models to study tip focused growth by means of endocytosis and exocytosis, cytoskeletal reorientation and reorganization and ion gradient modifications [1,2]. The *in vitro* pollen germination is very useful to study alterations associated with mutant phenotypes [3]. 

The study of pollen tube growth and guidance up to its target cells in the embryo sac has been the focus of several research works, and the need to faithfully simulate the *in vivo* system is always one of the principal objectives involved. Although more difficult to germinate *in vitro* than bicellular pollen grains, also true for other species with tricellular pollen, Arabidopsis pollen germination was convincingly established by Fan and coworkers in 2001. The pollen germination rate was nearly 75% and the average pollen tube length reached 135 µm after 6h of incubation [4]. Since then, a large number of the research work that involved pollen tube growth in Arabidopsis was performed using this growth medium. 

A few years ago, several studies showed that the pollen transcriptome was unique and that there was a decline in the number of diverse transcripts accompanied by pollen maturity together with an increase in the proportion of male gametophyte-specific transcripts [5,6,7]. Wang *et al*. [8] presented a study with the first genome-wide view of the transcriptome changes during the transition from mature pollen grains to growing pollen tubes in Arabidopsis, using the Fan *et al*. [4] growth medium. The authors concluded that transcription was increased during this process, both in terms of the total number of transcribed genes and of the transcriptional levels of some genes. 

In search of a robust method that could improve Arabidopsis pollen tube germination, Boavida and McCormick [9] developed another growth medium that substantially improved the *in vitro* performance of these pollen tubes. This protocol should facilitate both the functional analyses of insertion mutants affecting male gametophyte function, and the gene expression analysis during pollen tube growth [9]. Pollen tubes show a typical polar growth, migrating through the pistil tissues toward the ovules and it is known that interaction with the pistil renders pollen tubes competent to respond to guidance signals secreted by pistil specialized cells [10,11] and makes pollen tubes extend faster than growing *in vitro* [12]. For these reasons, Qin *et al*. [13], using a different pollen tube growth medium, showed that pollen tubes that had grown through the pistil tissues had a distinct gene expression profile and expressed a substantially larger fraction of the Arabidopsis genome than pollen grains or pollen tubes grown *in vitro*.

Very recently, another pollen tube microarray experiment was performed, in order to evaluate the role of two pollen-specific arabinogalactan proteins (AGPs) in pollen tube growth and development. In that experiment the Boavida and McCormick [9] pollen tube growth medium was used [14]. In the present work, we evaluated how these three different and well established pollen tube growth media affected wild type pollen tube growth performance and gene expression. Knowing how important for pollen tube growth and guidance is the delivery of new wall material [15] we evaluated some of the more representative pollen-specific AGP genes, as well as other cell wall-related enzyme genes (CEL3, EXPB5, BGLU12, GAUT 6, GAUT 13, PPME1 and SPS2F). The pollen tube wall has an organization consisting mainly of pectins mostly methylated at the apex and demethylated in the shank, together with cellulose and hemicellulose and cross-linked with hydroxyprolin-rich glycoproteins, including extensins and AGPs [16]. AGPs belong to a family of plant glycoproteins, whose members have hydroxyproline-rich protein backbones, are highly glycosylated, and are generally associated with the plasma membrane and cell wall, although they can also be found in the extracellular matrix. AGPs are ubiquitously expressed in plants, and many evidences suggest their involvement in all major cell wall-associated processes; most AGPs have a predicted C-terminal glycosylphosphatidylinositol (GPI) lipid anchor addition sequence, so it has been suggested that they may play a critical role in cell-cell signaling and cell recognition during pollination [17,18]. CEL3 (a cellulase) and β-glucosidase 12 are enzymes involved in hydrolyzing *O*-glycosyl compounds [19], while EXPB5 is an expansin that mediates cell wall extension in plants [20]. Pectin methylesterases catalyse the demethylesterification of cell wall polygalacturonans, and SPS2F which encodes a protein with putative sucrose-phosphate synthase activity, is involved in pollen exine formation [21]. 

The aim of the present work was to know to what extent the composition of Arabidopsis pollen germination media affected gene expression. Thus, a principal component analysis (PCA) was made with the existing wild type Arabidopsis pollen tube microarray data, and real-time PCR assays were performed with a collection of genes The results presented in this work may be particularly significant when assessing or designing Arabidopsis pollen tube transcriptome or proteome experiments.

## 2. Results and Discussion

Microarray experiments have recently been performed on Arabidopsis pollen tubes by different research teams, including our own [8,13,14]. Despite the fact that in those experiments the overall percentage of expressed genes was fairly coincident (approximately 30% of the total number of genes represented in the GeneChip Arabidopsis ATH1 Genome Array), there were some remarkable differences among the specific data sets of expressed genes. One possible variable that could have influenced the pattern of expression of *in vitro* cultured Arabidopsis pollen tubes was the growth media. In fact, the three published microarray experiments were done with pollen tubes germinated in different nutrient composition, and so we wanted to assess to what extent the composition of the growth medium influences the pattern of gene expression of germinating pollen tubes. To that end a series of parallel experiments were performed with pollen tubes grown in different growth media, Q, W and B, which stand for the media used by Qin *et al*. [13], Wang *et al*. [8] and Costa *et al*. [14], respectively, and set up a statistical analysis of relevant microarray data sets.

### 2.1. Quantitative qPCR Analysis of Selected Genes

#### 2.1.1. Arabinogalactan Protein Genes

**Figure 1:**
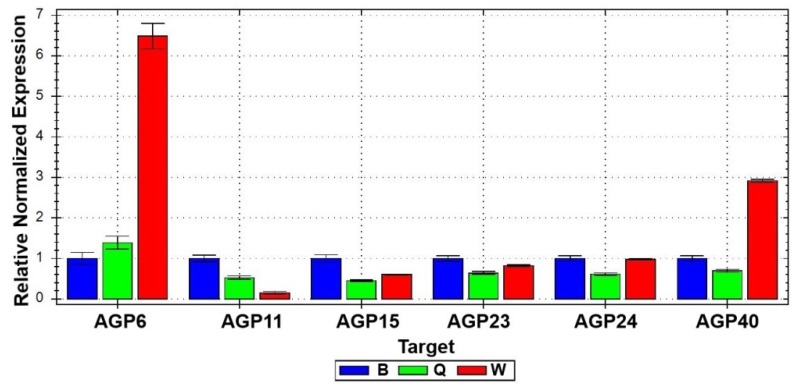
Real-time PCR results of the expression levels of six AGP genes in pollen tubes growing in three different growth media, B, Q and W.

AGPs are GPI-anchored cell wall associated glycoproteins that have been implicated in several important biological phenomena including stress and wounding responses [22]. The pollen tube of Arabidopsis has six AGPs expressed at very high levels [8], four of which are pollen-specific: AGP6, AGP11, AGP23 and AGP40. The search for the biological function of AGPs has been the subject of several investigations, and it has recently been shown that AGP6 and AGP11 are important for pollen tube cell wall assembly, elongation and correct germination time [3,18]. For this reason, in the present work, the six highly expressed AGP genes of the pollen tube were studied by Real-time quantitative RT-PCR (qPCR), using RNA preparations obtained from Arabidopsis pollen tubes germinated and grown in media B, Q and W. qPCR results were normalized using ACT4 and UBC9 as reference genes, and the results obtained with medium B were arbitrarily set as the control situation to which the levels of gene expression of the other media were compared. Transcript levels for most AGPs were notably lower in medium Q when compared with the other two media (Figure 1). In contrast, AGP6 and AGP40 were found to be overexpressed in medium W compared with media B and Q (Figure 1), which indicated that at the level of the AGP gene family, different genes respond differently to the culture medium formulation. 

#### 2.1.2. Cell Wall Related Genes

**Figure 2:**
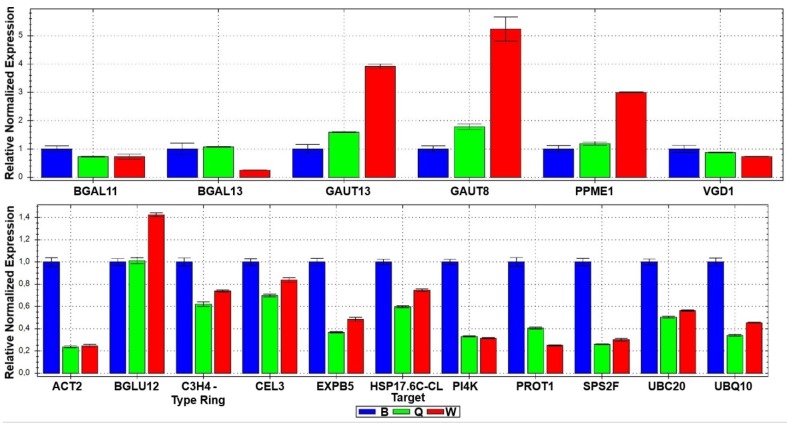
Real-time PCR results of the expression levels of seventeen cell wall related genes in pollen tubes growing in the three different growth media, B, Q and W.

Cell wall related enzymes with high expression levels in Arabidopsis pollen tubes where chosen namely CELLULASE 3 (CEL3), β-GLUCOSIDASE 12 (β-GLU12), GALACTURONOSYL TRANSFERASE 6 and GALACTURONOSYL TRANSFERASE 13 (GAUT6 and GAUT13), EXPANSIN B5 (EXPB5), and a few others associated with cell wall modifications and loosening for cell wall expansion, namely SUCROSE-PHOSPHATE SYNTHASE 2F (SPS2F) and PECTIN METHYLESTERASES (PPME1 and VGD1). These last three enzymes are involved more directly with the pollen cell wall building and with the pollen tube growth (Figure 2). 

CEL3 is an enzyme that hydrolyses *O*-glycosyl compounds of the cell wall allowing loosening of the cell wall, which in turn is essential for cell elongation. The family of glycosyl hydrolases (endo-β 1,4-glucanases) constitutes a group of enzymes that can hydrolyze internal linkages in β-1,4 glucan substrates. The most conspicuous of β-1,4 glucans present in plants is cellulose, which is the most important structural component of the cell wall. These genes are also referred to as cellulases. In Arabidopsis, this family consists of 25 members comprising a few membrane proteins that could be anchored at the plasma membrane and a large number of proteins with a predicted amino acid signal in the *N*-terminus that would direct secretion to the cell wall. A homozygous T-DNA insertion mutant that does not express *CEL5* was obtained and the modest consequence of abolishing *CEL5* expression suggested that there are multiple redundant genes regulating the process of sloughing of the root cap, including *CEL3*/At1g71380, a paralog of the *CEL5* gene that is also expressed in the root cap cells. Thus, these two endo-β-1,4-glucanases may have a role in the sloughing of border cells from the root tip. This is an important topic and AGPs are also supposed to be involved in plant defense in border-like cells [22]. In this experiment with pollen tubes, *CEL3* had a small expression level difference between the three growth media, but *BGLU12* had an increased expression in W medium compared to the others. 

Pectins are structurally complex plant cell-wall polysaccharides that contain 1,4-linked α-d-galactopyranosyluronic acid residues. Galacturonic acid (GalA) is the most abundant glycosyl residue in the three types of pectin present in all plant primary walls: homogalacturonans (HG), and type I and type II rhamnogalacturonans I. Many studies have shown that pectins contribute to the physical and biochemical properties of the wall and are required for normal plant growth and development. A complete understanding of pectin function requires knowledge of pectin biosynthetic enzymes and their corresponding genes [23].

Galacturonosyltransferases are enzymes that transfer galacturonic acid from uridine 5'-diphosphogalacturonic acid onto the pectic polysaccharide homogalacturonan [23]. Pectin and xylan polysaccharides are affected by the loss of galacturonosyltransferase function, as demonstrated by the altered galacturonic acid, xylose, rhamnose, galactose, and arabinose composition of distinct *gaut* mutant walls [24]. GAUT6 and GAUT13 showed a huge increase in the expression level when pollen tubes were growing in the W medium, when compared to B medium (Figure 2). 

Pectin is also the major component of pollen tube cell wall. The galacturonic acid is methylesterified at the Golgi and can be demethylesterified at the wall, by specific pectin methylesterases (PME). VANGUARD1 (VGD1) encodes a pollen-specific PME that is required for the normal growth of pollen tubes *in vivo* and the demethylesterification catalyzed by VGD1 is indeed critical for pollen tube growth [25]. In this work, PPME1 presented a significant increase in expression level in medium W when compared to B and Q media (Figure 2).

SPS2F is a sucrose-phosphate synthase that showed a decrease in expression level in W and Q growth media compared to B medium. In a mutant screen performed in order to find abnormal pollen exine structure, one of the *kaonashi* mutants identified was *kns2*. KNS2 was shown to be identical to SPS2F (At5g11110). The *kns2* mutant showed no visible phenotype other than exine abnormality, although this gene is expressed in many organs [21] (Figure 2).

Expansins are plant cell wall-loosening proteins that mediate pH-dependent extension of the cell wall. They are classified in two major groups, α-expansins (EXPA) and β-expansins (EXPB), and both are encoded by multigene families in land plants. The biological functions of α-expansins include cell enlargement, fruit softening and abscission [26], whereas β-expansin functions are not yet well established [20]. It was recently shown that new properties of grass pollen β-expansins would be to promote the penetration of the pollen tube through the stigma and style, most likely by weakening the middle lamella [27]. *EXPB5* is over expressed in medium W when compared to Q and even more expressed in medium B, when compared to the two others pollen tube growth media (Figure 2). 

#### 2.1.3. Statistical Analysis of Microarray Data

A Principal Components Analysis (PCA) was performed on published microarray data of *in vitro*-germinated Arabidopsis pollen tubes. The data included in the analysis consisted of the probes which had a present call in all three experiments. 

PCA compares the observations for the same probe (or gene) between samples, returning a value that estimates the degree of segregation between the observations. The cumulative effects of the deviation values allow the determination of the degree of separation between sets. The results of PCA are often represented by projections of scatter plots where the distance to the origin represents the degree of separation between the samples. Three axes summarize the values of the transposition vectors of the data sets. The more deviant an observation is relatively to its counterparts, the more pronounced will be its factor values (F1, F2 or F3) in relation to the origin [28,29].

**Figure 3:**
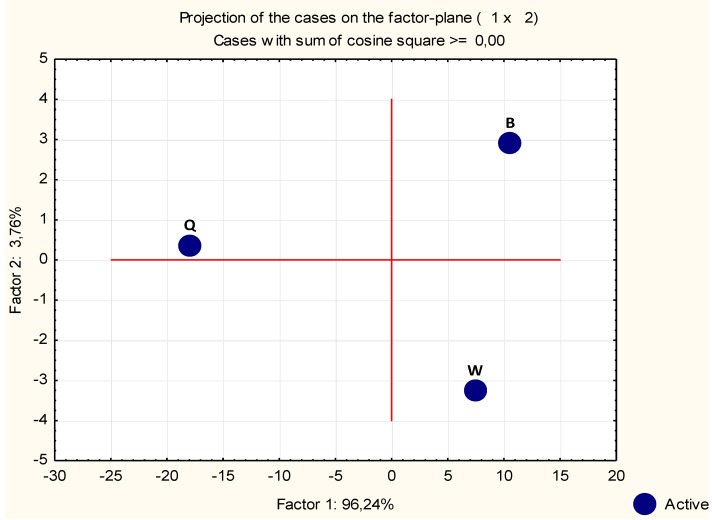
Principal Components Analysis (PCA) projection of three growth media normalized microarray probes values. The elements of this data set presented a scattered distribution, suggesting that the microarray results indeed diverge among them. Factor 1 axis shows the majority of the deviation, 96.24%, and Factor 2 axis contributes only with 3.76%. According to this PCA analysis the overall observation of B and W seem to be more closely related between them than with Q, however B and W also deviate in a smaller degree.

The three data sets do diverge among them. The projection view in Figure 3 represents the compilation of the deviations between data sets of the individual observations (probe signal values). Factor 1 axis contributed with almost the totality of the deviation (96.24%), while Factor 2 axis contributed with only a small part of the deviation (3.76%).

According to this PCA, the observations obtained with B and W growth media are more closely related to each other than to those obtained with Q.

In order to determine which of the probes/genes in the data sets were contributing mostly to the observed deviations, the eigenvalues were determined for all three data sets. All the probes that fell off the average values were identified using box plots.

The lists of the identified genes are shown in Supplementary Tables 1–3. A functional classification analysis was performed on each of those lists, and in all three a majority of genes was classified under hydrolase activity (in molecular function) and under cell wall (in cellular component) (Supplementary Figures 1–3).

#### 2.1.4. Pollen Tube Germination Rate and Length

**Figure 4:**
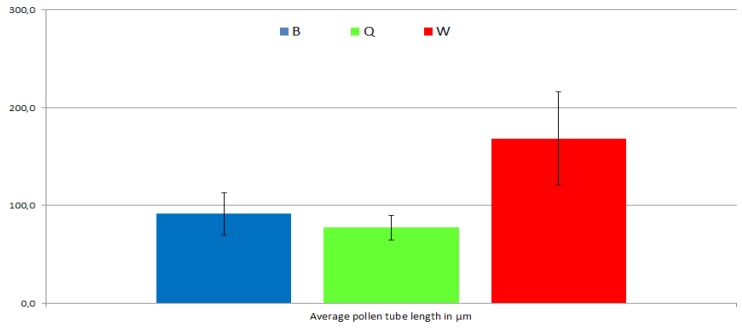
Wt pollen tube length after 6 hours of incubation in the three different growth media, B, Q and W.

The three different pollen tube media formulations (B, Q and W) were compared in parallel experiments for pollen tube germination rate, after 4 hours of incubation time, and also for tube length, after 6 hours of incubation time. Growth medium B produced a significantly higher germination rate (88%) when compared with media Q (52%) and W (49%). However, this difference was not reflected in average tube length, which was approximately double for medium W than that for media B and Q (Figure 4).

It certainly is not a straightforward matter to assign differences in growth rate or gene expression pattern to specific components or pH values of each growth medium, it became nevertheless apparent that differences in either pH or nutrient composition or both may have a deep impact on the biology of the pollen tube. Furthermore, Boavida and McCormick [9] tested culture media differing only in pH and observed significant shifts both in the percentage of germination and in average tube length, which would necessarily be associated with changes in gene expression patterns. These observations may be quite relevant for studies dealing with pollen tube germination, growth and physiology. 

## 3. Experimental Section

### 3.1. Plant Material and Growth Conditions

Arabidopsis thaliana ecotype Col-0 was obtained from the European Arabidopsis Stock Centre (NASC). Seeds were germinated and grown in Murashige and Skoog (MS) medium supplemented with 0.7% agar. The plantlets were transferred to soil 14 days after germination and kept in a growth chamber at 21 °C under long days (16 h light/8 h dark) and 60% relative humidity. Pollen was harvested only from freshly opened flowers corresponding to stages 13 to 15 as described in Smyth *et al*. [30], and as described in [9]. Briefly, 40 flowers were collected in 1.5 mL microtubes containing 1 mL of the respective growth medium and gently shaken. The flowers were then removed from the microtube, together with any visible tissue debris, and the pollen rich solution was then centrifuged for 6 min at 5,000 g. The pollen grain pellets were combined in groups of three to obtain a total of 120 flowers for each group. The combined preparation was resuspended in 750 µL of fresh growth medium, and finally transferred to flat bottom glass vials (20 mm diameter).

Three different culture media were tested, the composition of which is stated in Table 1. 

**Table 1:** Composition of the pollen germination media used in the present work.

	Medium W [8]	Medium Q [13]	Medium B [14]
Sucrose	19.8%	18.0%	10.0%
Lactalbumin hydrolysate	0.05%	-	-
Myo-inositol	10 mM	-	-
MES ^¶^	5 mM	-	-
CaCl_2_	5 mM	2 mM	5 mM
H_3_BO_3_	1.5 mM	1.5 mM	1.5 mM
KCl	1 mM	-	5 mM
MgSO_4_	0.8 mM	1 mM	1 mM
pH	5.8	7.0	7.4

^¶^ 2-(*N*-morpholino)ethanesulfonic acid.

### 3.2. RNA Extraction and Real-Time PCR

Six hour pollen tube cultures were washed through a 60 µm mesh nylon filters (Millipore NY6004700) with the respective growth medium in order to discard ungerminated pollen. The pollen tube-containing filters were processed for RNA extraction with Quiagen RNeasy plant kit according to manufacturer’s instructions, using RLT Lysis buffer supplemented with 5 µL of β-mercaptoethanol per 450 µL of lysis buffer.

The extracted RNA was reverse transcribed using Promega Reverse Transcription System and poly(dT)12-18 to prime the reactions. cDNA was amplified using the iQ™ SYBR^®^ Green Supermix on the iQ™5 Real-Time PCR Detection System (Biorad). Real-time PCR reactions were performed in duplicates. After an initial period of 3 min at 95 °C, each of the 40 PCR cycles consisted of a denaturation step of 10 s at 95 °C, an annealing step of 30 s at 56.4 °C and an extension step of 30 s at 72 °C. With each PCR reaction, a melting curve was obtained to check for amplification specificity and reaction contaminations, by heating the amplification products from 60 °C to 95 °C in 5 s intervals. Serial dilutions of pure genomic DNA from Arabidopsis ecotype Col-0 were used to verify primer efficiency. ACT4 (At5g59370) and UBC9 (At4g27960) were used as reference genes. These genes have been extensively used in our laboratory and were validated by the specific qPCR Biorad software. The sequences of the primers used in qPCR assays are stated in Supplementary Table 4. qPCR data and primer efficiency were analyzed with iQ5 2.0 Standard Edition Optical System Software v2.0.148.060623 (Biorad), using the Livak calculation method [31] for normalized expression using medium B as the control condition.

### 3.3. Pollen Tube Microarray Data Analysis

The data sets for the *in vitro* germinated wild type pollen tube were extracted from the available microarray databases associated to the respective publications [8,14,15]. All the probes with a present call simultaneously for all three data sets were considered and normalized by calculating the probe signal average value of the replicates in each data set. Using the statistics analysis software Statistica^®^ a PCA (principal components analysis) was performed to evaluate the degree of separation between the three data sets. The probes with the eigenvalues outside of the average were identified using the PCA axis information. The values for the identified probes on the three normalized data sets were then compared to the average value for that specific probe, thus identifying in which of the experimental conditions the probe value was more deviant. Finally, the GO annotations of the probes, contributing to the drift of each data set, were analyzed in order to determine if the different culture media would induce the differential expression of genes associated to dissimilar molecular functions or biological processes.

### 3.4. Pollen Tube Germination Rates and Length Measurement

After 4 h incubation period in darkness, pollen tubes were centrifuged at 5,000 g for 5 min and the pellet resuspended in 100 µL of culture medium. Five 20 µL samples were observed under the light microscope, and the germination rates determined by dividing the total number of pollen tubes observed by the total number of pollen grains. Standard deviation was calculated using the mean of the estimated germination rates.

Pollen tube elongation measurements were made after 6 hours in culture. The pollen tubes were centrifuged at 5,000 g for 5 min and the pollen tube rich pellets were observed with a light microscope. Pollen tube lengths were determined using the magnification adjusted measurement function of the Zeiss Axiovision (version 4.8.2.0) software. The average length and standard deviation were calculated using the mean of 3 replicates (n > 250).

## 4. Conclusions

These results indicate that the nutrient composition of the pollen germination media may influence pollen tube gene expression and metabolism to a significant extent and so caution must be taken when interpreting transcriptomic data of pollen tubes.

## Supplementary Files

Supplementary File 1
