# Supplementary material for: Growth Media Induces Variation in Cell Wall Associated Gene Expression in Arabidopsis thaliana Pollen Tube
Source: Plants (Basel). 2013 Jun 25;2(3):429–40. doi: 10.3390/plants2030429 (PMC4844376; doi:10.3390/plants2030429)

Supplementary Materials

Table S1. List of genes that were identified as principal contributors to the deviation observed in B over Q and W, in the PCA analysis.

Affy ID	AGI ID	Annotation (Lookup from 2010-12-20)—for multiple identifier mappings only the annotation for the first identifier is shown, except in the case of the multi-line output option
**245053_at**	At2g26450	Plant invertase/pectin methylesterase inhibitor superfamily
**245528_at**	At4g15530	PPDK__pyruvate orthophosphate dikinase
**245658_at**	At1g28270	RALFL4__ralf-like 4
**245700_at**	At5g04180	ACA3_ATACA3__alpha carbonic anhydrase 3
**245946_at**	At5g19580	glyoxal oxidase-related protein
**246123_at**	At5g20390	Glycosyl hydrolase superfamily protein
**246234_at**	At4g37280	MRG family protein
**246491_at**	At5g16100	unknown protein; Has 30201 Blast hits to 17322 proteins in 780 species: Archae, 12; Bacteria, 1396; Metazoa, 17338; Fungi, 3422; Plants, 5037; Viruses, 0; Other Eukaryotes, 2996 (source: NCBI BLink).
**246545_at**	At5g15110	Pectate lyase family protein
**246841_at**	At5g26700	RmlC-like cupins superfamily protein
**247512_at**	At5g61720	Protein of unknown function (DUF1216)
**247804_at**	At5g58170	GDPDL7_SVL5__SHV3-like 5
**247843_at**	At5g58050	GDPDL6_SVL4__SHV3-like 4
**248470_at**	At5g50830	unknown protein; Has 4750 Blast hits to 3160 proteins in 341 species: Archae, 14; Bacteria, 239; Metazoa, 1329; Fungi, 394; Plants, 197; Viruses, 79; Other Eukaryotes, 2498 (source: NCBI BLink).
**248534_at**	At5g50030	Plant invertase/pectin methylesterase inhibitor superfamily protein
**248581_at**	At5g49900	Beta-glucosidase, GBA2 type family protein
**248714_at**	At5g48140	Pectin lyase-like superfamily protein
**248822_at**	At5g47000	Peroxidase superfamily protein
**248926_at**	At5g45880	Pollen Ole e 1 allergen and extensin family protein
**249049_at**	At5g44340	TUB4__tubulin beta chain 4
**249402_at**	At5g40155	Defensin-like (DEFL) family protein
**249429_at**	At5g39880	unknown protein; FUNCTIONS IN: molecular_function unknown; INVOLVED IN: biological_process unknown; LOCATED IN: endomembrane system; EXPRESSED IN: 10 plant structures; EXPRESSED DURING: L mature pollen stage, M germinated pollen stage, 4 anthesis, C globular stage, petal differentiation and expansion stage; BEST Arabidopsis thaliana protein match is: unknown protein (TAIR:AT3G28750.1); Has 25 Blast hits to 25 proteins in 3 species: Archae, 0; Bacteria, 2; Metazoa, 0; Fungi, 0; Plants, 23; Viruses, 0; Other Eukaryotes, 0 (source: NCBI BLink).
**250154_at**	At5g15140	Galactose mutarotase-like superfamily protein
**250606_s_at**	At1g69940<br>At5g07410	ATPPME1_PPME1__Pectin lyase-like superfamily protein
**251250_at**	At3g62180	Plant invertase/pectin methylesterase inhibitor superfamily protein
**251252_at**	At3g62230	DAF1__F-box family protein
**251258_at**	At3g62170	VGDH2__VANGUARD 1 homolog 2
**251358_at**	At3g61160	Protein kinase superfamily protein
**251590_at**	At3g57690	AGP23_ATAGP23__arabinogalactan protein 23
**251804_at**	At3g55430	O-Glycosyl hydrolases family 17 protein
**252052_at**	At3g52600	AtcwINV2_CWINV2__cell wall invertase 2
**252085_s_at**	At3g52000<br>At3g52010	scpl36__serine carboxypeptidase-like 36
**252212_at**	At3g50310	MAPKKK20_MKKK20__mitogen-activated protein kinase kinase kinase 20
**252440_at**	At3g47440	TIP5;1__tonoplast intrinsic protein 5;1
**252710_at**	At3g43860	AtGH9A4_GH9A4__glycosyl hydrolase 9A4
**253153_at**	At4g35700	DAZ3__zinc finger (C2H2 type) family protein
**253526_at**	At4g31420	Zinc finger protein 622
**253599_at**	At4g30860	ASHR3_SDG4__SET domain group 4
**253660_at**	At4g30140	CDEF1__GDSL-like Lipase/Acylhydrolase superfamily protein
**254024_at**	At4g25780	CAP (Cysteine-rich secretory proteins, Antigen 5, and Pathogenesis-related 1 protein) superfamily protein
**254123_at**	At4g24640	APPB1__Plant invertase/pectin methylesterase inhibitor superfamily protein
**255479_at**	At4g02380	AtLEA5_SAG21__senescence-associated gene 21
**256277_at**	At3g12120	FAD2__fatty acid desaturase 2
**256285_at**	At3g12510	MADS-box family protein
**256584_at**	At3g28750	unknown protein; LOCATED IN: endomembrane system; EXPRESSED IN: 12 plant structures; EXPRESSED DURING: L mature pollen stage, M germinated pollen stage, 4 anthesis, C globular stage, petal differentiation and expansion stage; CONTAINS InterPro DOMAIN/s: Protein of unknown function DUF1216 (InterPro:IPR009605); BEST Arabidopsis thaliana protein match is: unknown protein (TAIR:AT5G39880.1); Has 37 Blast hits to 31 proteins in 5 species: Archae, 0; Bacteria, 0; Metazoa, 0; Fungi, 0; Plants, 28; Viruses, 0; Other Eukaryotes, 9 (source: NCBI BLink).
**256966_at**	At3g13400	sks13__SKU5 similar 13
**257392_at**	At2g24450	FLA3__FASCICLIN-like arabinogalactan protein 3 precursor

Table S1. *Cont.*

Affy ID	AGI ID	Annotation (Lookup from 2010-12-20)—for multiple identifier mappings only the annotation for the first identifier is shown, except in the case of the multi-line output option
**257469_at**	At1g49290	unknown protein; BEST Arabidopsis thaliana protein match is: unknown protein (TAIR:AT5G13620.1); Has 99 Blast hits to 93 proteins in 11 species: Archae, 0; Bacteria, 0; Metazoa, 2; Fungi, 0; Plants, 97; Viruses, 0; Other Eukaryotes, 0 (source: NCBI BLink).
**257809_at**	At3g27060	ATTSO2_TSO2__Ferritin/ribonucleotide reductase-like family protein
**257819_at**	At3g25165	RALFL25__ralf-like 25
**257886_at**	At3g17060	Pectin lyase-like superfamily protein
**257981_at**	At3g20770	AtEIN3_EIN3__Ethylene insensitive 3 family protein
**257986_at**	At3g20865	AGP40__arabinogalactan protein 40
**258077_at**	At3g26110	Anther-specific protein agp1-like
**258092_at**	At3g14595	Ribosomal protein L18ae family
**258279_at**	At3g26870	Plant self-incompatibility protein S1 family
**258280_at**	At3g26880	Plant self-incompatibility protein S1 family
**258326_at**	At3g22760	SOL1__Tesmin/TSO1-like CXC domain-containing protein
**258639_at**	At3g07820	Pectin lyase-like superfamily protein
**258748_at**	At3g05930	GLP8__germin-like protein 8
**258889_at**	At3g05610	Plant invertase/pectin methylesterase inhibitor superfamily
**259044_at**	At3g03430	Calcium-binding EF-hand family protein
**259189_at**	At3g01700	AGP11_ATAGP11__arabinogalactan protein 11
**259266_at**	At3g01240	unknown protein; FUNCTIONS IN: molecular_function unknown; INVOLVED IN: biological_process unknown; LOCATED IN: endomembrane system; EXPRESSED IN: 11 plant structures; EXPRESSED DURING: L mature pollen stage, M germinated pollen stage, 4 anthesis, C globular stage, petal differentiation and expansion stage; BEST Arabidopsis thaliana protein match is: unknown protein (TAIR:AT3G01230.1); Has 12 Blast hits to 12 proteins in 3 species: Archae, 0; Bacteria, 0; Metazoa, 0; Fungi, 0; Plants, 11; Viruses, 0; Other Eukaryotes, 1 (source: NCBI BLink).
**259269_at**	At3g01270	Pectate lyase family protein
**260386_at**	At1g74010	Calcium-dependent phosphotriesterase superfamily protein
**260847_s_at**	At1g17290<br>At1g72330	AlaAT1__alanine aminotransferase
**260888_at**	At1g29140	Pollen Ole e 1 allergen and extensin family protein
**261511_at**	At1g71770	PAB5__poly(A)-binding protein 5
**261623_at**	At1g01980	FAD-binding Berberine family protein
**262122_at**	At1g02790	PGA4__polygalacturonase 4
**262282_at**	At1g68610	PCR11__PLANT CADMIUM RESISTANCE 11
**262291_at**	At1g70790	Calcium-dependent lipid-binding (CaLB domain) family protein
**262393_at**	At1g49490	Leucine-rich repeat (LRR) family protein
**262571_at**	At1g15430	Protein of unknown function (DUF1644)
**262620_at**	At1g06540	unknown protein; BEST Arabidopsis thaliana protein match is: unknown protein (TAIR:AT2G30930.1); Has 16 Blast hits to 16 proteins in 3 species: Archae, 0; Bacteria, 0; Metazoa, 0; Fungi, 0; Plants, 16; Viruses, 0; Other Eukaryotes, 0 (source: NCBI BLink).
**262676_at**	At1g75950	ASK1_ATSKP1_SKP1_SKP1A_UIP1__S phase kinase-associated protein 1
**262696_at**	At1g75870	unknown protein; Has 10 Blast hits to 10 proteins in 5 species: Archae, 0; Bacteria, 0; Metazoa, 0; Fungi, 0; Plants, 10; Viruses, 0; Other Eukaryotes, 0 (source: NCBI BLink).
**262760_at**	At1g10770	Plant invertase/pectin methylesterase inhibitor superfamily protein
**262885_at**	At1g64740	TUA1__alpha-1 tubulin
**263043_at**	At1g23350	Plant invertase/pectin methylesterase inhibitor superfamily protein
**263215_at**	At1g30710	FAD-binding Berberine family protein
**263681_at**	At1g26840	ATORC6_ORC6__origin recognition complex protein 6
**265007_s_at**	At1g61563<br>At1g61566	RALFL8__ralf-like 8
**265022_at**	At1g24520	BCP1__homolog of Brassica campestris pollen protein 1
**265080_at**	At1g55570	sks12__SKU5 similar 12
**265127_at**	At1g55560	sks14__SKU5 similar 14
**265960_at**	At2g37470	Histone superfamily protein
**266750_s_at**	At2g47030<br>At2g47040	VGDH1__Plant invertase/pectin methylesterase inhibitor superfamily
**266764_at**	At2g47050	Plant invertase/pectin methylesterase inhibitor superfamily protein
**266977_at**	At2g39420	alpha/beta-Hydrolases superfamily protein
**267590_at**	At2g39700	ATEXP4_ATEXPA4_ATHEXP ALPHA 1.6_EXPA4__expansin A4

Table 2. List of genes that were identified as principal contributors to the deviation observed in Q over B and W, in the PCA analysis.

Affy ID	AGI ID	Annotation (Lookup from 2010-12-20)—for multiple identifier mappings only the annotation for the first identifier is shown, except in the case of the multi-line output option
**246099_at**	At5g20230	ATBCB_BCB_BCB_SAG14__blue-copper-binding protein
**246208_at**	At4g36490	ATSFH12_SFH12__SEC14-like 12
**246751_at**	At5g27870	Plant invertase/pectin methylesterase inhibitor superfamily
**246761_at**	At5g27980	Seed maturation protein
**247512_at**	At5g61720	Protein of unknown function (DUF1216)
**248037_at**	At5g55930	ATOPT1_OPT1__oligopeptide transporter 1
**248194_at**	At5g54095	unknown protein; FUNCTIONS IN: molecular_function unknown; INVOLVED IN: N-terminal protein myristoylation; LOCATED IN: cellular_component unknown; EXPRESSED IN: 9 plant structures; EXPRESSED DURING: L mature pollen stage, M germinated pollen stage, 4 anthesis, C globular stage, petal differentiation and expansion stage; BEST Arabidopsis thaliana protein match is: unknown protein (TAIR:AT4G27580.1); Has 30201 Blast hits to 17322 proteins in 780 species: Archae, 12; Bacteria, 1396; Metazoa, 17338; Fungi, 3422; Plants, 5037; Viruses, 0; Other Eukaryotes, 2996 (source: NCBI BLink).
**248758_at**	At5g47620	RNA-binding (RRM/RBD/RNP motifs) family protein
**249447_at**	At5g39400	ATPTEN1_PTEN1__Calcium/lipid-binding (CaLB) phosphatase
**249536_at**	At5g38760	Late embryogenesis abundant protein (LEA) family protein
**250358_at**	At5g11740	AGP15_ATAGP15__arabinogalactan protein 15
**250608_at**	At5g07420	Pectin lyase-like superfamily protein
**250917_at**	At5g03690	Aldolase superfamily protein
**251228_at**	At3g62710	Glycosyl hydrolase family protein
**251258_at**	At3g62170	VGDH2__VANGUARD 1 homolog 2
**252085_s_at**	At3g52000<br>At3g52010	scpl36__serine carboxypeptidase-like 36
**252710_at**	At3g43860	AtGH9A4_GH9A4__glycosyl hydrolase 9A4
**252820_at**	At3g42640	AHA8_HA8__H(+)-ATPase 8
**254001_at**	At4g26260	MIOX4__myo-inositol oxygenase 4
**254716_at**	At4g13560	UNE15__Late embryogenesis abundant protein (LEA) family protein
**256385_at**	At1g66580	RPL10C_SAG24__senescence associated gene 24
**256580_s_at**	At3g28810<br>At3g28820	Protein of unknown function (DUF1216)
**256581_at**	At3g28830	Protein of unknown function (DUF1216)
**256588_at**	At3g28790	Protein of unknown function (DUF1216)
**256955_at**	At3g13390	sks11__SKU5 similar 11
**257077_at**	At3g19690	CAP (Cysteine-rich secretory proteins, Antigen 5, and Pathogenesis-related 1 protein) superfamily protein
**257090_at**	At3g20530	Protein kinase superfamily protein
**257121_at**	At3g20210<br>At3g20220	DELTA-VPE_DELTAVPE__delta vacuolar processing enzyme
**257532_at**	At3g04700<br>At3g04710	Protein of unknown function (DUF1685)
**257736_at**	At3g27410	unknown protein; Has 10 Blast hits to 10 proteins in 2 species: Archae, 0; Bacteria, 0; Metazoa, 0; Fungi, 0; Plants, 10; Viruses, 0; Other Eukaryotes, 0 (source: NCBI BLink).
**257821_at**	At3g25170	RALFL26__ralf-like 26
**258058_at**	At3g28980	Protein of unknown function (DUF1216)
**258498_at**	At3g02480	Late embryogenesis abundant protein (LEA) family protein
**258639_at**	At3g07820	Pectin lyase-like superfamily protein
**258645_s_at**	At3g07850<br>At3g14040	Pectin lyase-like superfamily protein
**258677_at**	At3g08730	ATPK1_ATPK6_ATS6K1_PK1_PK6_S6K1__protein-serine kinase 1
**258680_at**	At3g08580	AAC1__ADP/ATP carrier 1
**259064_at**	At3g07490	AGD11_AtCML3_CML3__ARF-GAP domain 11
**262664_at**	At1g13970	Protein of unknown function (DUF1336)
**262885_at**	At1g64740	TUA1__alpha-1 tubulin
**263062_at**	At2g18180	Sec14p-like phosphatidylinositol transfer family protein
**263144_at**	At1g54070	Dormancy/auxin associated family protein
**264923_s_at**	At1g60740<br>At1g65970	Thioredoxin superfamily protein
**264993_at**	At1g67290	GLOX1__glyoxal oxidase-related protein
**265404_at**	At2g16730	BGAL13__glycosyl hydrolase family 35 protein
**266029_at**	At2g05850	scpl38__serine carboxypeptidase-like 38
**266428_at**	At2g07180	Protein kinase superfamily protein
**266592_at**	At2g46210	AtSLD2_SLD2__Fatty acid/sphingolipid desaturase
**266765_at**	At2g46860	AtPPa3_PPa3__pyrophosphorylase 3
**266918_at**	At2g45800	PLIM2a__GATA type zinc finger transcription factor family protein
**267590_at**	At2g39700	ATEXP4_ATEXPA4_ATHEXP ALPHA 1.6_EXPA4__expansin A4

Table 3. List of genes that were identified as principal contributors to the deviation observed in W over B and Q, in the PCA analysis.

Affy ID	AGI ID	Annotation (Lookup from 2010-12-20)—for multiple identifier mappings only the annotation for the first identifier is shown, except in the case of the multi-line output option
**245700_at**	At5g04180	ACA3_ATACA3__alpha carbonic anhydrase 3
**245946_at**	At5g19580	glyoxal oxidase-related protein
**245956_s_at**	At5g28540<br>At5g42020	BIP1__heat shock protein 70 (Hsp 70) family protein
**246099_at**	At5g20230	ATBCB_BCB_BCB_SAG14__blue-copper-binding protein
**246118_at**	At5g20340	BG5__beta-1,3-glucanase 5
**246631_at**	At1g50740	Transmembrane proteins 14C
**248038_at**	At5g55980	serine-rich protein-related
**248470_at**	At5g50830	unknown protein; Has 4750 Blast hits to 3160 proteins in 341 species: Archae, 14; Bacteria, 239; Metazoa, 1329; Fungi, 394; Plants, 197; Viruses, 79; Other Eukaryotes, 2498 (source: NCBI BLink).
**249049_at**	At5g44340	TUB4__tubulin beta chain 4
**249187_at**	At5g43060	Granulin repeat cysteine protease family protein
**249243_s_at**	At5g42260<br>At5g44640	BGLU12__beta glucosidase 12
**249375_at**	At5g40730	AGP24_ATAGP24__arabinogalactan protein 24
**249584_s_at**	At5g37810<br>At5g37820	NIP4;1_NLM4__NOD26-like intrinsic protein 4;1
**250174_at**	At5g14380	AGP6__arabinogalactan protein 6
**250631_at**	At5g07430	Pectin lyase-like superfamily protein
**251250_at**	At3g62180	Plant invertase/pectin methylesterase inhibitor superfamily protein
**253226_at**	At4g35010	BGAL11__beta-galactosidase 11
**253814_at**	At4g28290	unknown protein; FUNCTIONS IN: molecular_function unknown; INVOLVED IN: biological_process unknown; LOCATED IN: chloroplast; EXPRESSED IN: 23 plant structures; EXPRESSED DURING: 15 growth stages; Has 45 Blast hits to 45 proteins in 11 species: Archae, 0; Bacteria, 0; Metazoa, 0; Fungi, 0; Plants, 45; Viruses, 0; Other Eukaryotes, 0 (source: NCBI BLink).
**253831_at**	At4g27580	unknown protein; FUNCTIONS IN: molecular_function unknown; INVOLVED IN: biological_process unknown; LOCATED IN: mitochondrion, cell wall; EXPRESSED IN: 9 plant structures; EXPRESSED DURING: 6 growth stages; Has 30201 Blast hits to 17322 proteins in 780 species: Archae, 12; Bacteria, 1396; Metazoa, 17338; Fungi, 3422; Plants, 5037; Viruses, 0; Other Eukaryotes, 2996 (source: NCBI BLink).
**255515_at**	At4g02250	Plant invertase/pectin methylesterase inhibitor superfamily protein
**256277_at**	At3g12120	FAD2__fatty acid desaturase 2
**259866_at**	At1g76640	Calcium-binding EF-hand family protein
**260401_at**	At1g69840	SPFH/Band 7/PHB domain-containing membrane-associated protein family
**260943_at**	At1g45145	ATH5_ATTRX5_LIV1_TRX5__thioredoxin H-type 5
**262022_at**	At1g35490	bZIP family transcription factor
**263126_at**	At1g78460	SOUL heme-binding family protein
**263950_at**	At2g36020	HVA22J__HVA22-like protein J
**264655_at**	At1g09070	(AT)SRC2_SRC2__soybean gene regulated by cold-2
**266428_at**	At2g07180	Protein kinase superfamily protein

Table 4. List of primers used in the qPCR assay.

AGI ID	Primer ID	Forward primer	Reverse primer
At4g27960	UBC9	GTTTCACCACCCTTTCTTC	AAATCCCACGATCAAATTCC
At5g59370	ACT4	CACCAAACGAAGACGGAAGT	CATTGGGAGTGGAAATTGTTT
At1g71380	CEL3	TCTCCAAGTCATTGCTCTTCTTCC	CGTTGTCTCCTGCGTCATAGTAC
At3g60570	EXPB5	GCAAACGGTGATGGGAACTTCG	GGACACGGCGGAGGTAAGC
At5g11110	SPS2F	TGGTGGTGTTCGTGGGAGATTCAG	TTAGCCTCGGTGATGTTGGGACTG
At5g42260	BGLU13	CCATTGGGACACACCGCAAAGC	ACACCTTCCTGGAGCCATTACACC
At3g02870	VTC4	GAGACATTCTGATGGACCTTACG	TCCGAGACTTAACGATTGATTGG
At5g14380	AGP6	TTCTAAGTTAAGTCGTCCAC	GACATTTAGGTTTATATTTACTCC
At3g01700	AGP11	CCACGTAATGTCAAGC	CAACAGGGGATGATGCTTTC
At3g57690	AGP23	AATGGAGATGAAGAAGATTG	TGCAAGTAGTAGCTGAAG
At5g40730	AGP24	CGGTTACAGATGGAGGAGGCACTC	AGGACACCACACGTAGCCACAATC
At3g20865	AGP40	GCGGCTACAATGGAGTCTC	CAAGAAGGCGGAGAGTGATG
AT4G35010	BGAL11	GGAATGGGAAAAGGGTTGAT	GCTCAGGCTTCACATTAGGC
AT2G16730	BGAL13	AAACAAGGCGGTTTGAACAC	GGAAGTCCTCCATGAGTCCA
AT3G01040	GAUT13	GAATTTTGCTGGGTTGTCGT	ACAACATCACCTCCCTACGC
AT1G06780	GAUT6	CGGCTTAAGAGCATCACTCC	CCATCACTTTCAGCAGACGA
At3g18780	ACT2	GTGCTGGATTCTGGTGATGGTGTG	CGTTGTCTCCTGCGTCATAGTAC
At4g33240	PI4K	TCCTCCAGCCACACGCAGAATTG	GAAGCATAGCGGAAGCAAGCAACC
At5g60250	C3HC4-Type ring	CCATTGGGACACACCGCAAAGC	ACACCTTCCTGGAGCCATTACACC
AT5G12030	HSP17.6C-CL	GGCAAACGCACCCGCTATG	TTCACCTCTTCCTTCCTCAGTCC
At2g39890	PROT1	ATGGCGAGAGGCGGGTAC	GTGGTTGGCTAGAATGAATGTGAG
At1g50490	UBC20	AGATCCTCCGGCGTCTAATGG	TGCTTCCTGTTATTGTCCCTTTCC
At1g50490	UBQ10	GCTCCGACACCATTGACAAC	ACGCAGGACCAAGTGAAGAG
AT1G69940	PPME1	ATGCCTACACCGAGATGACC	AGTTTACAAAGCGGGTGGTG
AT2G47040	VGD1	ACTGGCCCAAGTCATTCAAC	CCACCGAGACCACAACTTTT

Figure 1. Functional classification of the genes listed in Table S1 that produced the most diverging transcript levels. Most of the genes are associated to cell organization and biogenesis, and developmental processes. Most of them are extracellular or located at the cell wall, and the most conspicuous functional annotation relates to hydrolase activity.

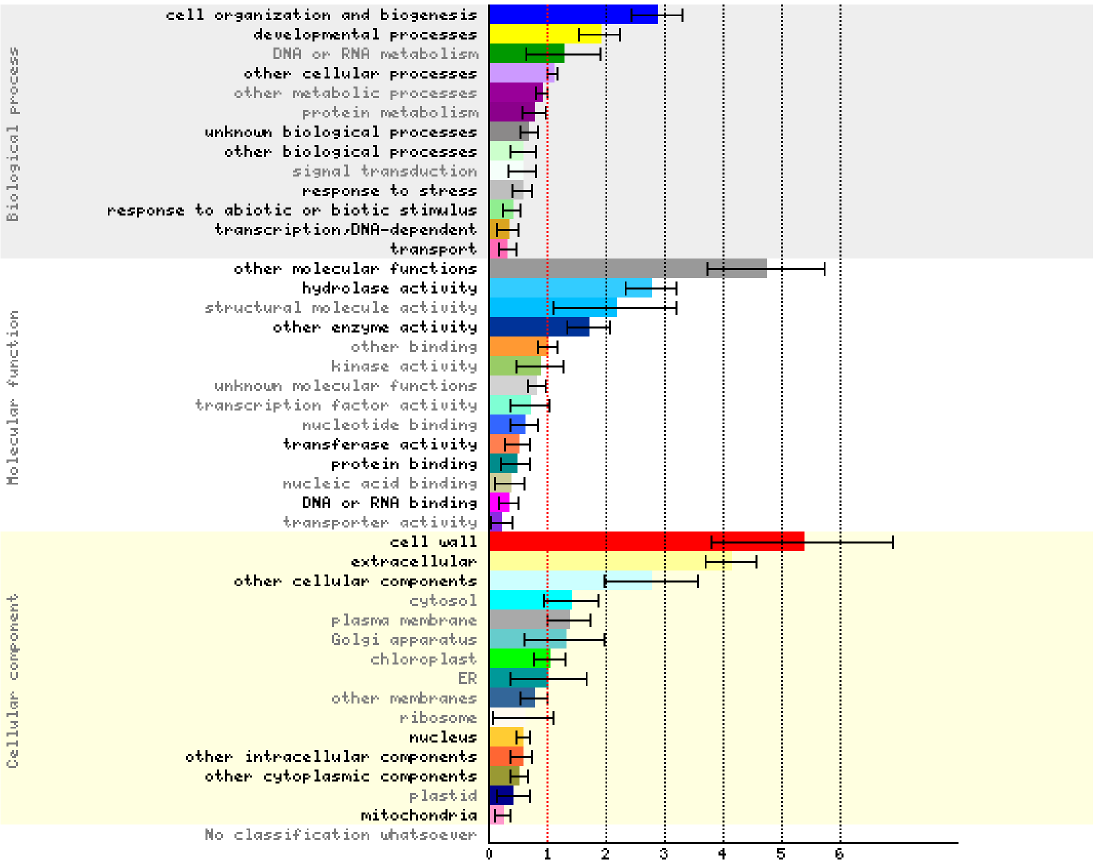

Figure 2. Functional classification of the genes listed in Table 2 that produced the most diverging transcript levels. Most of the genes are associated to cell organization and biogenesis, protein metabolism and developmental processes, most of them are located at the cell wall or are extracellular, and the most conspicuous functional annotation relates to hydrolase activity, transporter and other molecular functions.

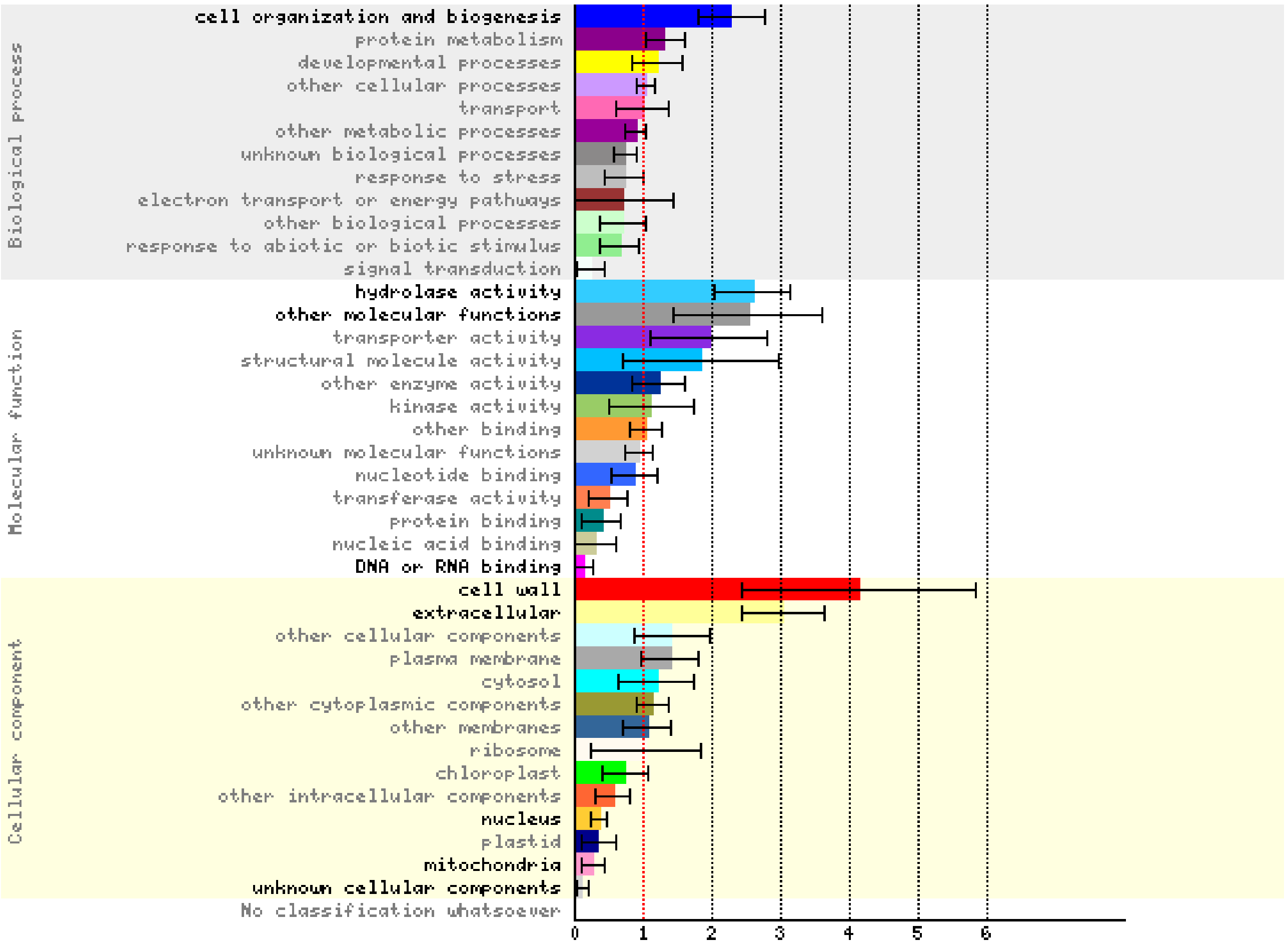

Figure 3. Functional classification of the genes listed in Table 3 that produced the most diverging transcript levels. Most of the genes are associated with cell organization and biogenesis, response to biotic and abiotic stimulus, response to stress and transport, most of them are extracellular or located at the Endoplasmic reticulum and cell wall, and the most conspicuous functional annotation relates to hydrolase activity.

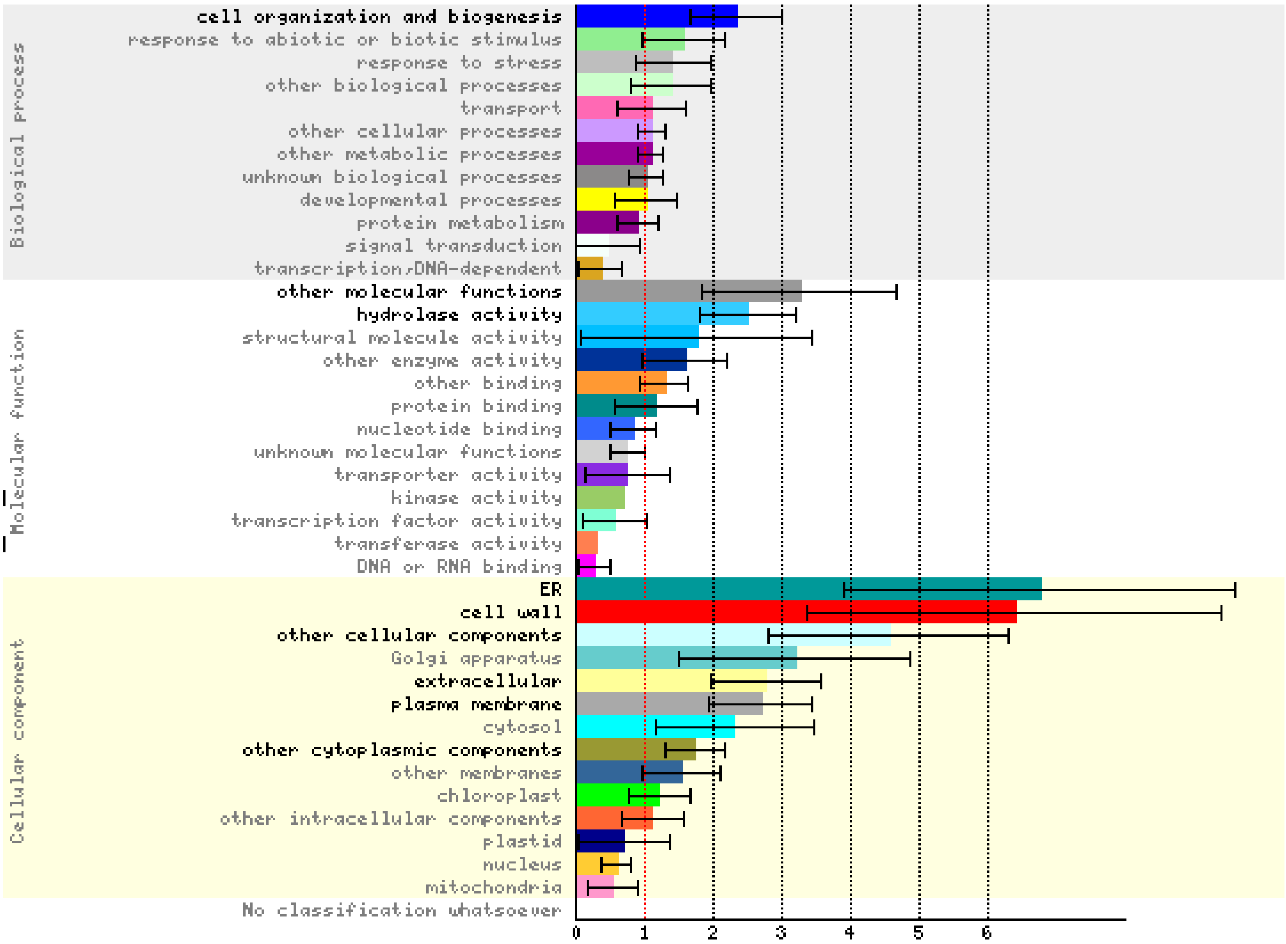

Supplement: Supplementary File 1 [file plants-02-00429-s001.pdf]
